# Traumatic brain injury induces long-lasting changes in immune and regenerative signaling

**DOI:** 10.1371/journal.pone.0214741

**Published:** 2019-04-03

**Authors:** Deborah R. Boone, Harris A. Weisz, Hannah E. Willey, Karen E. O. Torres, Michael T. Falduto, Mala Sinha, Heidi Spratt, Ian J. Bolding, Kathea M. Johnson, Margaret A. Parsley, Douglas S. DeWitt, Donald S. Prough, Helen L. Hellmich

**Affiliations:** 1 Department of Anesthesiology, University of Texas Medical Branch, Galveston, Texas, United States of America; 2 GenUs Biosystems, Northbrook, Illinois, United States of America; 3 Paradise Genomics, Inc., Northbrook, Illinois, United States of America; University of Florida, UNITED STATES

## Abstract

There are no existing treatments for the long-term degenerative effects of traumatic brain injury (TBI). This is due, in part, to our limited understanding of chronic TBI and uncertainty about which proposed mechanisms for long-term neurodegeneration are amenable to treatment with existing or novel drugs. Here, we used microarray and pathway analyses to interrogate TBI-induced gene expression in the rat hippocampus and cortex at several acute, subchronic and chronic intervals (24 hours, 2 weeks, 1, 2, 3, 6 and 12 months) after parasagittal fluid percussion injury. We used Ingenuity pathway analysis (IPA) and Gene Ontology enrichment analysis to identify significantly expressed genes and prominent cell signaling pathways that are dysregulated weeks to months after TBI and potentially amenable to therapeutic modulation. We noted long-term, coordinated changes in expression of genes belonging to canonical pathways associated with the innate immune response (i.e., NF-κB signaling, NFAT signaling, Complement System, Acute Phase Response, Toll-like receptor signaling, and Neuroinflammatory signaling). Bioinformatic analysis suggested that dysregulation of these immune mediators—many are key hub genes—would compromise multiple cell signaling pathways essential for homeostatic brain function, particularly those involved in cell survival and neuroplasticity. Importantly, the temporal profile of beneficial and maladaptive immunoregulatory genes in the weeks to months after the initial TBI suggests wider therapeutic windows than previously indicated.

## Introduction

There is a singular disquieting fact about traumatic brain injury (TBI); thus far, all successful preclinical studies have failed to translate [1, 2], indicating a serious disconnect between bench and bedside. The lack of approved treatments for TBI’s degenerative effects and the knowledge that TBI can be incurred by individuals at any time should be troubling to all. As one of the major causes of mortality and disability across all age groups in all countries, a recent review in the Lancet suggests that the world-wide incidence is estimated to be over 50 million cases a year with a cost to the global economy of approximately $400 billion a year [3]. It is imperative that we identify underlying pathogenic mechanisms that may be targeted with existing or novel drugs.

Another reason for urgency in identifying long-term pathogenic mechanisms is that TBI increases the risk of dementia later in life [4]. In a nationwide cohort study of several hundred thousand TBI survivors and controls in Sweden, the authors reported a time- and dose-dependent risk of developing dementia more than 30 years after TBI [5]. TBI is a risk factor for all neurological disorders associated with dementia such as Alzheimer’s, Parkinson’s and Huntington’s diseases [6] as well as Chronic Traumatic Encephalopathy [7] and psychiatric disorders such as depression [8]. Altogether, these chronic brain disorders represent some of the greatest threats to human health worldwide [9]. However, despite well documented lifelong deficits [10] and evidence for long lasting pathological changes after TBI [11], with few exceptions [12], there is a notable lack of studies on the molecular mechanisms of chronic neurodegeneration in animal models. Given that progressive neurodegeneration is the common factor across most human brain diseases, identifying the causal mechanisms in TBI would have major implications for the treatment of all these brain disorders.

While acknowledging the dismal state of TBI therapeutics, how do we address this challenge and provide hope for existing TBI survivors? What are the endogenous deleterious and protective signals in the injured brain that could be therapeutically targeted with currently approved drugs to improve recovery and functional outcome after TBI? Is it too late, or are there wider therapeutic windows that provide hope for current chronic TBI survivors? As part of a larger project documenting proteomic, histological, behavioral [13] and genomic changes occurring across five acute and chronic post-injury intervals (24 hours, 2 weeks, 3, 6 and 12 months post-TBI) in a rat model of TBI, we sought answers to these questions by analyzing the acute and chronic TBI-induced transcriptional profiles of two functionally connected brain regions—the hippocampus and cortex—that are associated with learning and memory and executive function [14–16].

Our previous studies [17, 18] led us to hypothesize that persistent injury-induced genomic changes in key hub genes belonging to essential cell signaling pathways contribute to long-term neurodegeneration. We recognized that there are clinically used drugs known to target some of these dysregulated signaling pathways. In this study, we found prominent dysregulation of several disease-associated canonical pathways in both hippocampus and cortex; notably, we have evidence of chronic dysregulation of pathways essential for cell survival, neuroplasticity and protein homeostasis (proteostasis) [19]. As our focus was identifying potentially druggable pathways, and there are multiple drugs that target inflammatory signaling, we noted with great interest that in both brain regions, genes in inflammatory and cell death pathways are acutely and chronically dysregulated after TBI.

## Materials and methods

### Animals

Adult, male Sprague-Dawley rats (250 g–300 g) from vendor Charles Rivers (Portland, Maine) were housed two per cage with food and water *ad libitum* in a vivarium with these constant conditions: light cycle (6: 00–18: 00) temperature (21°C–23°C), and humidity (40%–50%). All animal experiments were approved by the Institutional Animal Care and Use Committee of the University of Texas Medical Branch, Galveston, Texas and conducted according to the National Institutes of Health Guide for the Care and Use of Laboratory Animals (8^th^ edition, National Research Council). For each post-TBI interval, we analyzed gene expression in hippocampus and cortex of four naïve, four sham-injured and four TBI rats; thus, 84 rats were used for microarray and PCR array analysis.

### Surgical preparation

Rats were anesthetized with isoflurane in an anesthetic chamber, intubated, and mechanically ventilated with 1.5%-2.0% isoflurane in O_2_: room air (70:30) using a volume ventilator (EDCO Scientific, Chapel Hill, NC). Rats were prepared for parasagittal fluid-percussion injury (FPI) as previously described [17, 20]. Surgical preparation of sham-injured and TBI rats was identical except for the injury. Rats were placed in a stereotaxic head frame and the scalp was sagittaly incised. A 4.0 mm diameter hole was trephined into the skull 2.0 mm to the right of the sagittal suture and midway between lambda and bregma. A modified Luerlok syringe hub was placed over the exposed dura, bonded in place with cyanoacrylic adhesive and covered with dental acrylic. Isoflurane was discontinued; rats were connected to the fluid-percussion trauma device and immediately after the return of a withdrawal reflex to paw pinch a severe (2.3 atm) FPI; was administered. After FPI or sham injury, rats were disconnected from the fluid-percussion device and the time to right (the righting reflex) was measured. Rats were then placed back on isoflurane (2%), wound sites were infused with bupivicaine and sutured with prolene. Isoflurane was discontinued and the rats were extubated and allowed to recover in a warm, humidified incubator.

### Microdissection of hippocampus and cortex

Tissue dissections were performed using sterile, RNase-treated iris tissue forceps. The two cerebral hemispheres of the cortex were opened along the cerebral longitudinal fissure and separated with the forceps to expose the hippocampus. Each hippocampus was individually separated from the cortex and midbrain using the same Iris tissue forceps and each were placed into separate sterile Eppendorf tubes containing RNA*later* (ThermoFisher) and stored at 4°C or placed into separate empty sterile Eppendorf tubes and fresh frozen on dry ice for 3–5 minutes. After retrieval of the hippocampi, a small “snip” of cortex near the injury site was excised using the Iris forceps and placed into separate sterile Eppendorf tubes containing RNA*later* (ThermoFisher) and stored at 4°C or placed into separate empty sterile Eppendorf tubes and fresh frozen on dry ice for 3–5 minutes.

### Microarray analysis

Microdissected hippocampal and cortical tissue, collected at 24 hours, 2 weeks, 3, 6 and 12 months after FPI, were placed in a 1 ml tube and stored in RNA*later* (Ambion, Thermo Fisher) at 4°C. Tissue was shipped to GenUs Biosystems (Northbrook, IL) for microarray analysis. We have a long standing collaboration with GenUs scientists, who participated in the first MicroArray Quality Control (MAQC) study that demonstrated the inter- and intraplatform reproducibility of gene expression measurements [21]. Thus, our microarray procedures follow the recommended guidelines of the first and second MAQC studies [21, 22]. RNA was extracted and purified using Ribopure (Ambion), and total RNA samples were quantified by UV spectrophotometry (OD260/280). The concentration and quality of total RNA was assessed using an Agilent Bioanalyzer with the RNA6000 Pico Lab Chip (Agilent Technologies). First and second strand cDNA was prepared from the total RNA samples. Complementary RNA (cRNA) targets were prepared from the DNA template and verified on the Agilent Bioanalyzer. 1 μg of purified cRNA was fragmented to uniform size and hybridized to Agilent Rat GE 8x60K arrays (028279). Agilent Whole Rat Genome microarrays are comprised of approximately 41,000 60-mer probes designed to conserved exons across the transcripts of targeted genes. These probes represent well annotated, full length and partial human gene sequences from major public databases. Arrays were hybridized at 65°C for 17 hours in a rotating incubator and washed at 37°C for one minute. After staining with Streptavidin-Alexa555, rinsed and dried, arrays were scanned with an Agilent G2565 Microarray Scanner (Agilent Technologies, Santa Clara, CA) at 5 μm resolution. Agilent Feature Extraction software was used to process the scanned images from arrays (gridding and feature intensity extraction) and the data generated for each probe on the array was analyzed with GeneSpring GX v7.3.1 software (Agilent Technologies, Santa Clara, CA). To compare individual expression values across arrays, raw intensity data from each gene was normalized to the median intensity of the array. Genes were removed for further analysis if at least one replicate sample was not above background intensity. Further filtering was performed to only include genes whose values were within 50% for biological replicate samples.

### Bioinformatic analyses with Ingenuity pathway analysis, gene ontology, and Qlucore Omics Explorer

The filtered gene list was queried for genes differentially expressed (fold-change ≥1.4) in TBI relative to sham treatment. Statistically significant gene expression data (p < 0.05) from the GeneSpring analysis were uploaded into Ingenuity Pathway Analysis (IPA) software (QIAGEN). Core expression analysis was performed for genes 24 hours, 2 weeks, 3, 6, and 12 months post-injury, to identify critical canonical pathways in which genes were enriched and upstream genes that could potentially be master regulators. P-values were calculated based on a Fisher's Exact Test by considering the number of genes that comprised a given process or pathway within our dataset and the number known to be associated with that process in IPA's reference set. The more genes that overlapped in our dataset with the reference set, the more likely the association was not due to random chance. Thus, significant canonical pathways represent processes with an over representation of focus genes from our dataset greater than were expected to show up by chance. Gene Ontology enrichment analysis was used to identify top biological processes associated with differentially expressed genes at each post-injury interval. Qlucore Omics Explorer (QOE, Qlucore, Lund, Sweden), a bioinformatics software platform (based on R) that allows a dynamic and interactive visualization of multivariate data by projecting high dimensional data down to lower dimensions, was used for principal component analysis. Microarray data have been deposited for public access in the National Center for Biotechnology Information Gene Expression Omnibus under accession number GSE111452.

### Quantitative real-time PCR validation of microarray results

For validation of gene expression from 24 h, 2 wk, 3 mo, 6 mo, and 1 year microarray results, qRT-PCR was completed using total RNA isolated at GenUs Biosystems. For each post-injury interval, 10–20 differentially expressed genes from the microarray analysis were selected based on the IPA analysis. Total RNA (500 ng) was reversed-transcribed using the High Capacity Kit (Applied Biosystems) following manufacturer’s protocols. Q-PCR was performed on a Roche Light Cycler 96 using Taqman probes (Applied Biosystems) following manufacturer’s protocols. All data collected was analyzed using the Roche light cycler software, a data analysis tool for sample comparison using the ΔΔCT method for calculating the relative quantification of gene expression.

### 1 month and 2 month hippocampal and cortex 96 well custom qPCR arrays

#### RNA isolation, cDNA synthesis and qPCR

For custom qPCR array analysis of one and two month post-TBI rat brains, total RNA was isolated from microdissected hippocampal and cortex tissue using Trizol (Invitrogen) according to manufacturer’s protocol and DNase treated for 30 min at 37°C using Turbo DNase (Ambion). Total RNA (700 ng) was reverse transcribed using the iScript Reverse Transcription Kit (Bio-Rad) according to manufacturer’s protocol. QPCR was performed on a CFX384 Real-Time System (Bio-Rad) using a Sybr-green 384 well custom PCR array plate containing 4 individual 96-well gene groups (86 selected genes, 5 endogenous control genes, 5 plate controls). The thermo-profile cycling protocol: 2 min @ 95C for 1 cycle, 5 sec @ 95°C and 30 sec @ 60°C for 45 cycles and a melt curve 5 sec/step @ 65°C to 95°C for 1 cycle was used to run PCR plates. Data was collected and imported into the CFX Manager PrimePCR analysis software. Data was normalized to 5 endogenous control genes (Actb, Hprt1, Ldna, Rpl13a, and Rplp1) and PrimePCR plate controls were analyzed to verify RNA quality and to test performance of reactions. The ΔΔCT method for calculating the relative quantification of gene expression was used to calculate fold changes comparing sham and TBI groups to naïve groups. We previously employed a similar experimental design using pathway-specific PCR arrays to analyze gene expression in laser captured dying and surviving neurons [23].

### Experimental design and statistical analysis

**Fig 1:**
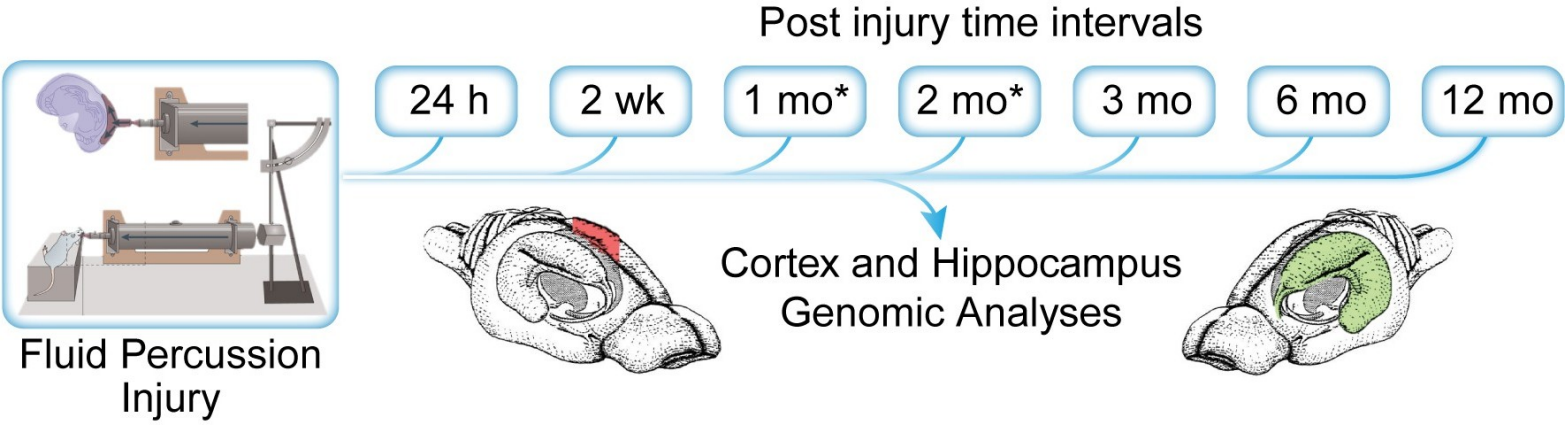
Schematic of experimental design. Rats were subjected to fluid percussion injury (FPI) and survived for 24 hours, 2 weeks, 3 month, 6 month, and 12 months. At the time of sacrifice, whole hippocampal and cortex tissue (beneath the injury site) were manually dissected and stored in *RNALater* at 4° C until processed for genomic analysis. (* 1 and 2 month time points were subsequently added to the experimental design after the initial analysis was completed for animals survived up to 1 year post-FPI).

The microarray analysis was initially performed on microdissected rat hippocampal and frontal cortex tissues from post-TBI intervals 24 hr, 2 weeks, 3, 6 and 12 months (Fig 1). To fill in gaps in gene expression between 2 weeks and 3 months, custom PCR array analysis was subsequently performed with microdissected hippocampal and cortex tissues collected from rats one and two months post-TBI. Microarray and PCR arrays for each post-injury interval were performed with four independent biological replicates each for naïve controls, sham-injured controls and TBI.

For statistical analysis of qPCR validation experiments, a Mann-Whitney test was performed to compare differences between Sham and TBI (p<0.05). For one and two months PCR arrays, normalized expression values were log transformed to distribute data normally. A one-way ANOVA was performed on each gene. A Benjamini-Hochberg (BH) correction test was run and a Tukey’s post hoc test was performed on the genes that had a BH q-value of <0.05.

## Results

### Experimental design of microarray and custom PCR array analyses

The model of parasagittal fluid-percussion injury (FPI) used in our studies recapitulates many important, clinically relevant hallmarks of human TBI [24] and has been shown by our group to produce measurable neurobehavioral deficits up to 12 months after injury [25]. Our original experimental design involved whole genome microarray analysis of microdissected rat hippocampal and frontal cortex tissues from post-TBI intervals 24 hr, 2 weeks, 3, 6 and 12 months (Fig 1). Subsequent analysis indicated a need for data from one and two months for validation of earlier (24 hr and 2 week) gene expression trends identified in our initial data analyses. Thus, the one and two month TBI rat experiments were performed a year after the original set of experimental rats and selected gene expression changes were assessed using custom PCR arrays comprised of biologically relevant genes derived from bioinformatics analysis of our microarray data. For the following description of our findings, descriptions of the bioinformatics analyses methods are confined to specific applications in IPA or QOE.

### Temporal gene expression profiling shows that injury-induced genomic changes persist up to a year after TBI

**Fig 2:**
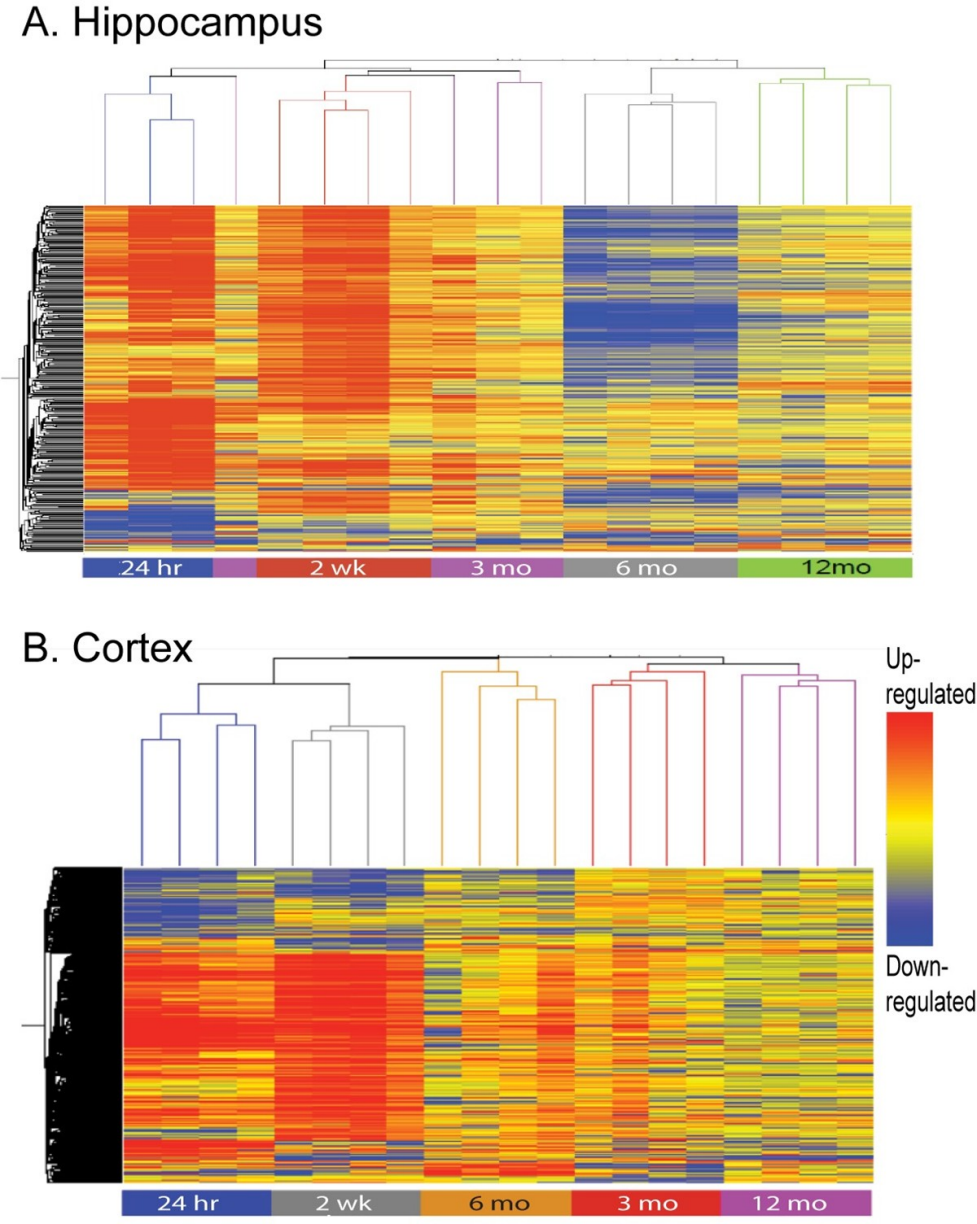
Hierarchical clustering of acute, subchronic and chronic gene expression after traumatic brain injury. Heatmaps of temporal (24 hour, 2 week, 3, 6,12 months) gene expression profiles of rat hippocampus (A) and cortex (B) after fluid percussion injury reflect the post-injury intervals.

Agilent microarray gene expression data for sham and TBI were normalized to the mean expression of the time/age-specific naïve samples. For clarity and to illustrate temporal gene expression (GE) patterns in the TBI groups, hierarchical clustering heatmaps were generated to show post-injury interval-specific GE across all hippocampus (Fig 2A) and cortex samples (Fig 2B). Hierarchical clustering, which groups genes with similar transcriptional profiles into clusters, revealed that temporal hippocampal GE profiles reflect the post-TBI survival time of each animal cohort and the trends are similar in the cortex except that 6 month GE profiles appear closer to 2 week profiles. The majority of injury-induced changes occur within the first 24 hours to two weeks but some genes or the cellular pathways comprised of these genes remain dysregulated up to 12 months. The heatmaps also show that the temporal hippocampal and cortex expression profiles are distinct, reflecting differences in TBI-induced gene expression in these two functionally connected brain regions that subserve different as well as common cognitive domains [26, 27].

**Fig 3:**
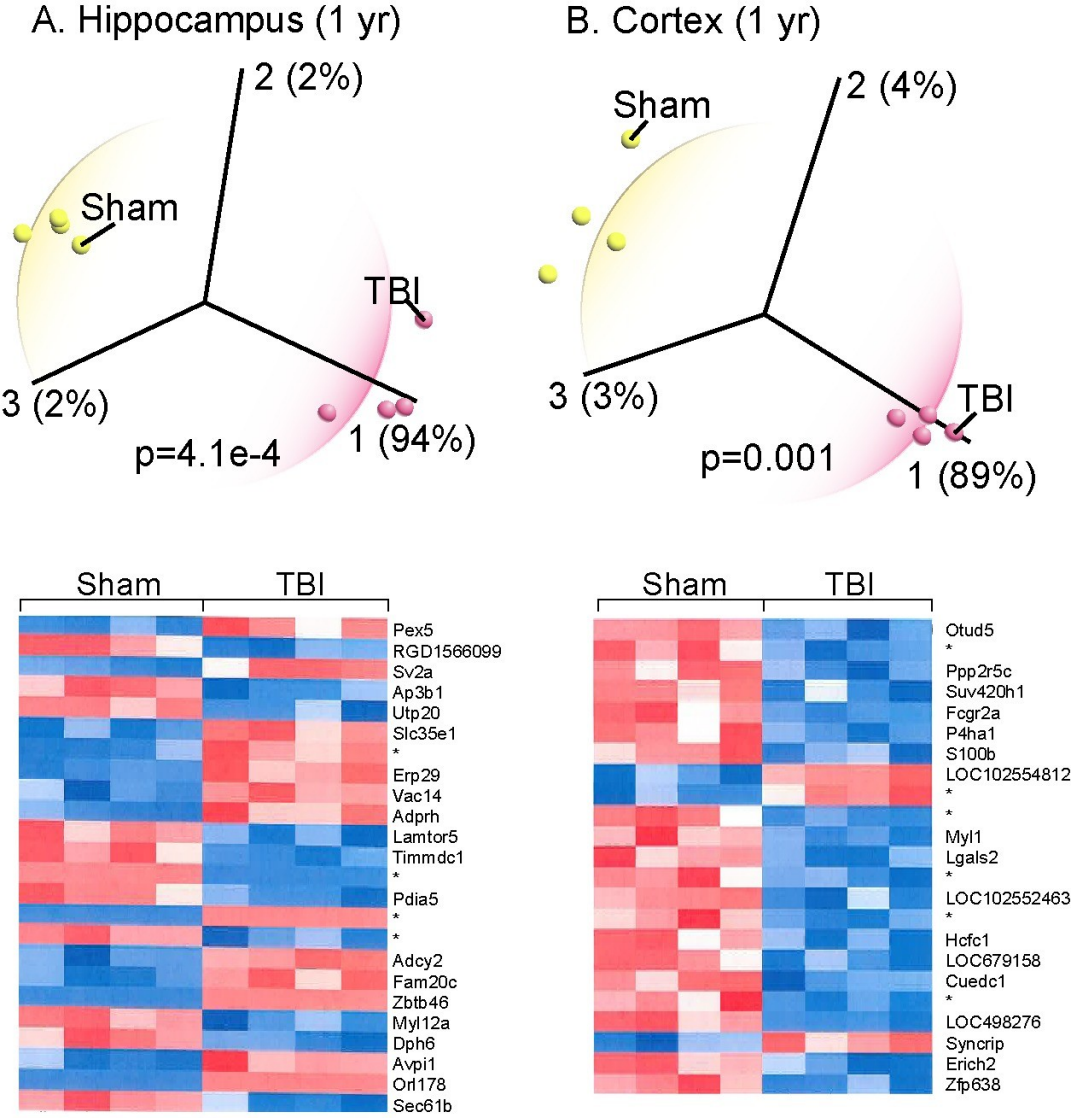
Prinicipal component analysis (PCA) of chronic gene expression one year post-TBI. Qlucore Omics Explorer was used to generate the PCA plots and heatmaps displaying genes that distinguish TBI from sham controls in the hippocampus (A) and cortex (B) at one year post-TBI. Annotated genes are associated with metabolism, synaptic function and proteostasis. Discriminating genes that are significantly differentially expressed but lack annotation (i.e. unknown and uncharacterized) are marked with an asterisk (*).

Qlucore Omics Explorer is a data analysis tool that combines powerful statistical and mathematical tools with instant visualization of large datasets with dimension reduction. The instant, interactive principal component analysis (PCA)- a data dimension reduction technique that allows visualization of high-dimensional, multivariate data without the loss of more information than necessary- performed in QOE is an unsupervised (i.e. no information about the experimental groups is used in the analysis) approach that is well suited for this time series gene expression study. Principle component analysis of hippocampal and cortical GE data shows that at each post-injury interval and as long as one year after injury, the TBI rats were clearly distinguishable from sham-injured controls (Fig 3A and Fig 3B; PCA plots of post-injury intervals 24 hr– 6 months for hippocampus and cortex are shown in Supporting Information, S1 Fig and S2 Fig). For the one year post-injury hippocampal and cortical data, the heatmaps display the differentially expressed genes that discriminate sham from the TBI groups in the PCA plots; the top three principal components shown in Fig 3A and Fig 3B capture 98% or 96%, respectively, of the variance in the GE data that separates the sham from TBI rats. Since a significant proportion of genes expressed in the mammalian brain are functionally enigmatic and lack annotation [28], it was not altogether surprising to find that some of the discriminating, significantly expressed genes (marked with asterisk in Fig 3 heatmaps) were unknown. For the rest, *in silico* analysis showed that the genes that distinguished sham from TBI rats were associated primarily with immune response, synaptic function and proteostasis and all annotated genes are known to be essential for normal homeostatic brain function [29].

### Ingenuity pathway analysis provides key insights into biological mechanisms of acute, subchronic and chronic gene expression

**Fig 4:**
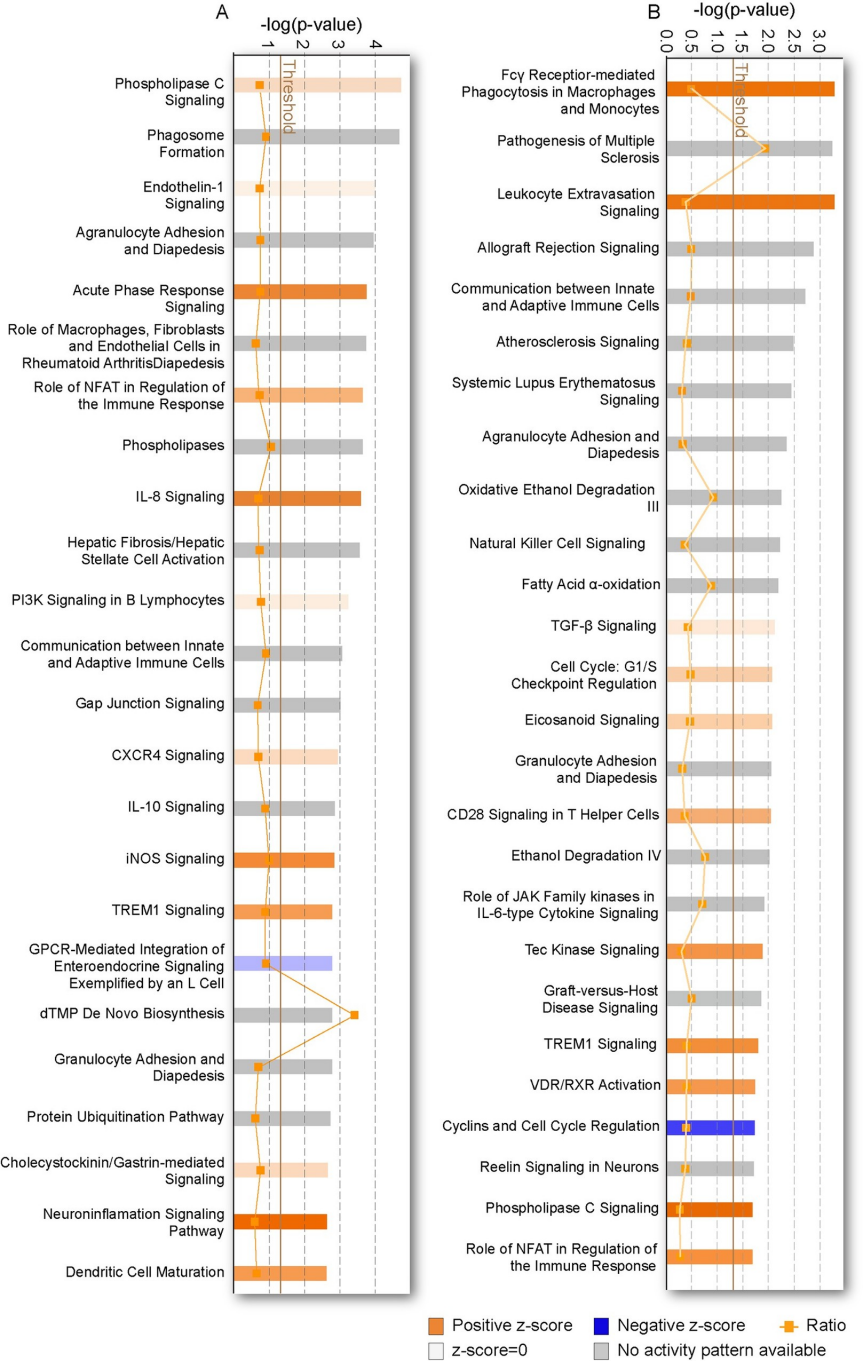
Ingenuity pathway canonical pathway analysis. Genes in the top 25 canonical pathways (complete list of pathways are shown in S1 File and S2 File) in the hippocampus (A) and cortex (B) at 24 hours post-TBI are prominently associated with inflammation and other immune related functions. Threshold indicates minimum significance level [–log (p-value) from Fisher’s exact test]. Ratio refers to the number of molecules from the dataset that map to the pathway listed divided by the total number of molecules that define the canonical pathway from within the IPA knowledgebase.

To gain mechanistic insights into the acute, subchronic and chronic gene expression changes after TBI, we performed bioinformatic analysis using IPA to interrogate and infer the biological relevance of perturbed gene networks. Using IPA facilitates a systems biology approach [30, 31] that has allowed us to interrogate functional gene networks rather than individual genes in isolation [17, 18, 32]. Genes imported into IPA had a stringency filter set for statistically significant genes (absolute fold-change >1.4, *p*-value ≤ 0.05) from across all time points in the ipsilateral hippocampus and cortex in TBI vs sham-injured controls. Biologically relevant changes were assessed in IPA through core expression analysis done for each set of filtered genes, at each discrete time point. The top 25 canonical pathways (CPs) at 24 hr for hippocampus and cortex are shown (Fig 4A and Fig 4B. Genes from all significant CPs and from each respective post-injury interval are listed in Supporting Information S1 File for hippocampus and S2 File for cortex); the greater the numbers of genes above the threshold in each pathway, there is a greater likelihood that the representative cell signaling pathway is functionally relevant to TBI pathogenesis. These analyses revealed sets of genes within and across time points in hippocampal and cortical tissue that were highly enriched in immune-related function and cell survival related canonical pathways.

**Fig 5:**
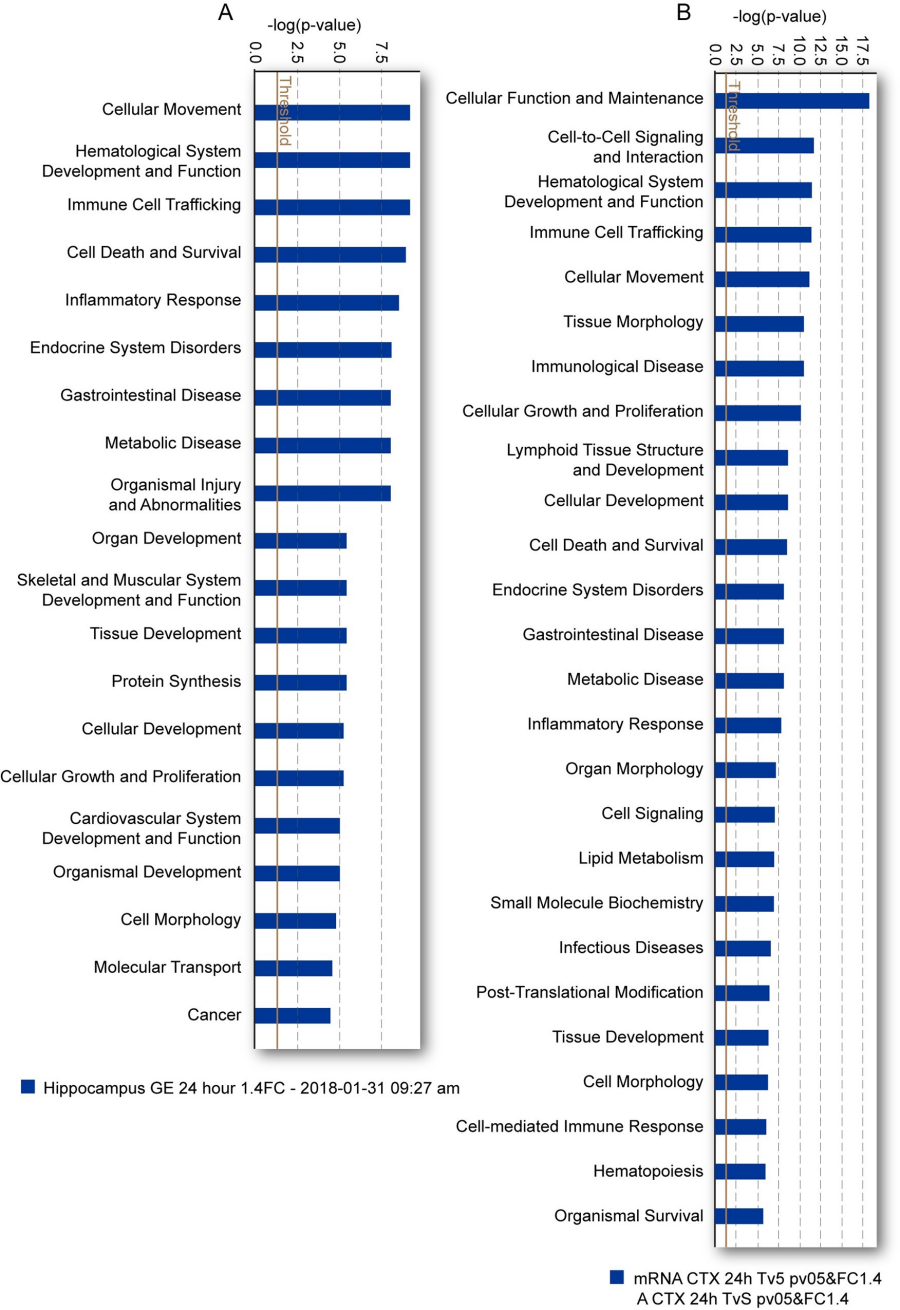
Ingenuity pathway diseases and functions analysis. Genes in the top 25 diseases and functions pathways (complete lists of pathways are shown in S3 File and S4 File) in the hippocampus (A) and cortex (B) at 24 hours post-TBI are prominently associated with inflammation and immune-related functions. Threshold indicates minimum significance level [–log (p-value) from Fisher’s exact test].

In concordance with previous transcriptome profiling studies of TBI in animal models [33, 34], we found that the injured rat hippocampus and cortex shared pathways associated with inflammation and immune response, neuropathic pain, cell death and apoptotic signaling among others. In addition, analysis of significant diseases and functions pathways in IPA which displays predicted cellular processes and biological functions based on the gene expression profiles, showed considerable overlap between the hippocampus and cortex (Fig 5A and Fig 5B, genes from all significant pathways are listed in Supporting Information S3 File and S4 File) but also showed differences that likely reflect the region-specific response to TBI. Again, we found a preponderance of immune-related functions represented by significantly expressed genes.

Gene Ontology enrichment analysis confirmed that differentially expressed genes in the hippocampus and cortex were significantly enriched in biological processes associated with immune response and inflammatory signaling (Supporting Information S5 File [GO hippocampus] and S6 File [GO cortex]). Overall, our temporal GE data corroborates the findings of acute TBI-induced genomic changes from other studies in rodent models [35] and our recent studies [17] and extends these findings to several subchronic and chronic post-injury intervals.

### Quantitative real time PCR validation of microarray results

Quantitative real-time PCR is an accepted validation tool for confirmation of differentially expressed genes obtained from microarray analysis. QPCR was completed for selected genes (*p*-value ≤ 0.05) that were enriched in canonical pathways relevant to TBI pathophysiology (Supporting Information S3 Fig for hippocampus and S4 Fig for cortex). In the hippocampus, qPCR was performed using TaqMan probes for 46 differentially expressed genes (significantly expressed genes at each post-injury interval were selected) to validate results of the microarray analyses. We confirmed trends in gene expression for 44/46 (95.6%) and found that 61% were significantly different between TBI and sham controls. We also confirmed that a small subset of these genes appeared differentially expressed across other post-injury intervals. In the cortex, qPCR was performed using Taqman probes to 70 differently expressed genes (significant at different post-injury intervals) to validate results of the microarray analyses. We confimed trends in gene expression for 61/70 (87%) and found 41% were significantly different between TBI and sham controls. Variability in qPCR data could be due to factors such as alternative RNA splicing and even with careful consideration of the variability in biological and technical procedures which could affect the correlation of microarray and qPCR data, the best correlation between the two techniques is around 0.8 [36]. Moreover, the original MicroArray Quality Control (MAQC) study (Genus Biosystems scientists were one of the original study groups that contributed to this study, MAQC Consortium, Nature Biotechnology, 2006) showed that at best, there was about 80–90% concordance in results from multiple microarray platforms [21, 22]. Based on these previous reports, we are confident that our qPCR reflects biologically relevant injury-induced gene expression changes. Thus, despite the limited number of biological replicates in our study for each experimental group, our finding that 95.6% and 87% of gene expression trends in the hippocampus and cortex, respectively, could be confirmed by qPCR indicated that microarray results did indeed reflect actual TBI-induced biological differences.

### Post-injury-interval gene expression reflects acute and persistent immune dysregulation

In seeking to derive biologically meaningful gene expression trends across all post-injury intervals, it is helpful to determine what are the shared common features in dysregulated genes that belong to identified pathways. In a recent study, a mathematical framework that was developed to understand the essential parameters in high dimensional gene expression data showed that modularity and low dimensionality which is a feature of this type of data allows an accurate extraction of transcriptional programs [37]; in other words, since a small number of features can accurately represent a signal comprised of a much larger number of features, data analysis tools such as IPA can accurately capture and represent biologically relevant programs from high dimensional microarray data. Across all acute (24 hr), subchronic, (2 weeks) and chronic (3, 6, 12 months) post-injury intervals, we found prominent dysregulation of multiple immune response and inflammatory cell signaling pathways (see Supporting Information S1–S4 Files).

**Fig 6:**
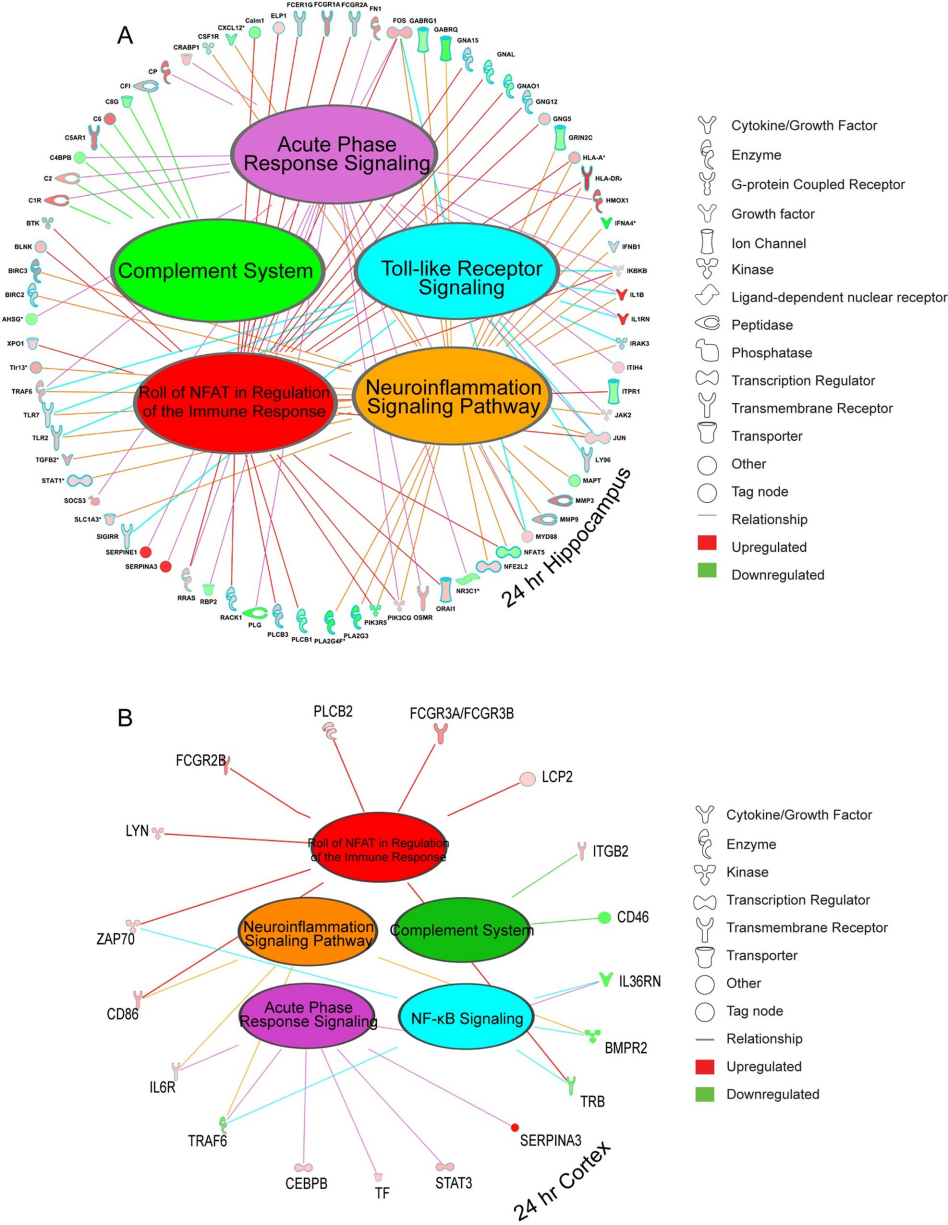
Dysregulated genes after TBI at 24 hr are associated with multiple immune-related pathways. Ingenuity Pathway Analysis shows that differentially expressed (DE) genes in the hippocampus (A) and to a lesser extent in cortex (B) overlap in five key immune-related pathways. Many of the DE genes are well known hub genes in essential cell signaling pathways.

Significantly dysregulated genes in the hippocampus at 24 hr were enriched in immune function related pathways, including “Neuroinflammation Signaling Pathway”; “NFAT Regulation of the Immune Response”; “Acute Phase Response Signaling”; “Toll-like Receptor (TLR) Signaling”; and, “Complement System”. In the cortex, NF-κB signaling was more prominent than TLR signaling. We used IPA’s Path designer tool to represent these five immune related pathways and their connection to differentially expressed TBI-associated genes in the hippocampus and cortex (Fig 6A and Fig 6B). Although overlapping pathways were affected in both brain regions, many more genes in these pathways were acutely affected by TBI in the hippocampus at 24 hr compared to the cortex.

**Fig 7:**
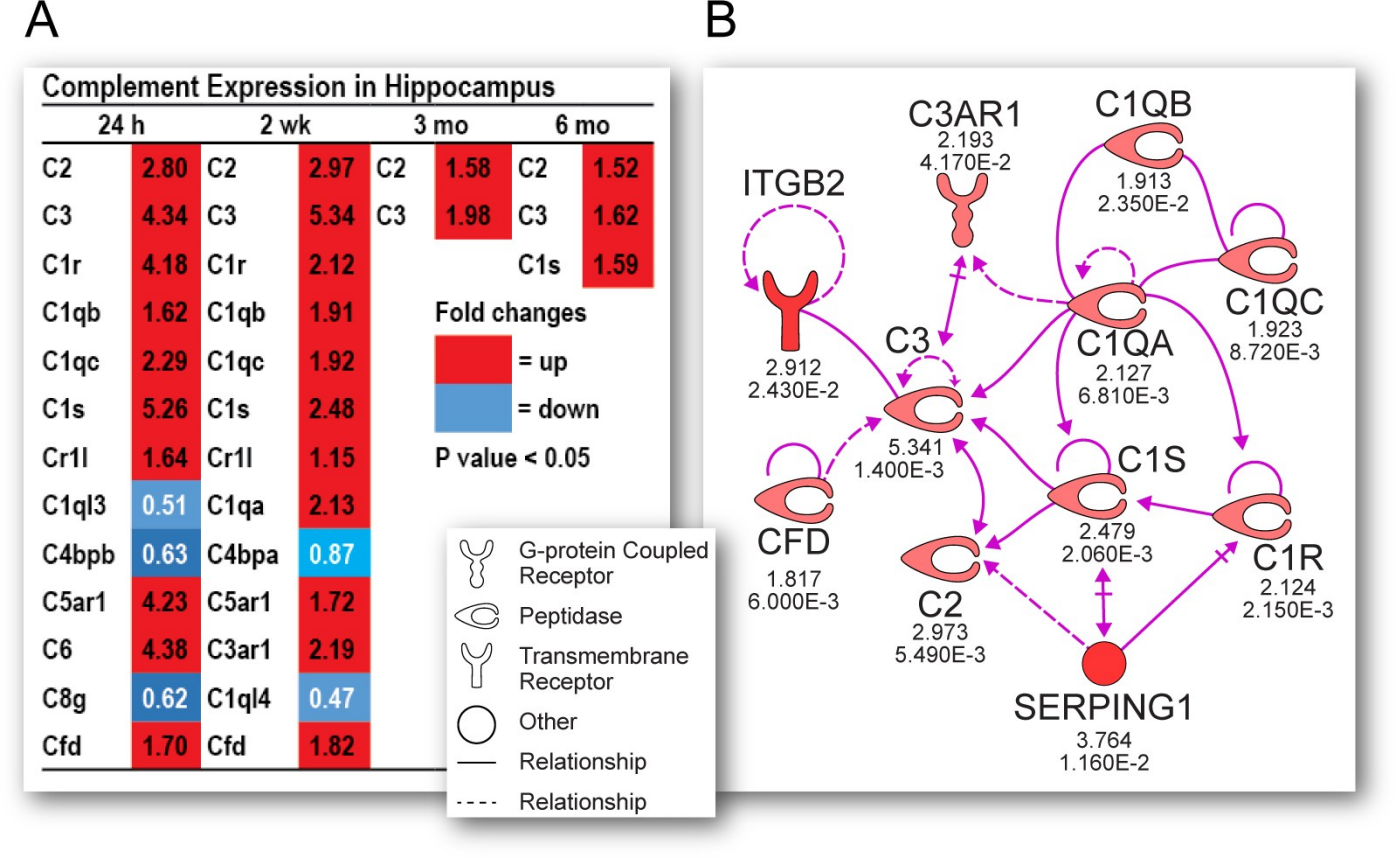
Acute and chronic complement expression in hippocampus. Complement genes are persistently dysregulated up to six months post-TBI (A). Ingenuity Pathway Analysis (IPA) Path designer graphics integrates contextual information and biological insights from IPA’s knowledge base to depict a coordinated dysregulation of complement genes at 24 hours post-TBI (B).

One great advantage of IPA is the access to the curated literature that underlies the functional annotation of identified genes and pathways. Thus, IPA analysis showed that many genes in these immune-related pathways are hub genes that are essential components of key cell signaling networks. One prominent set of genes that have been shown in preclinical studies to effect acute outcomes after TBI are members of the complement system [38]. The role of complement in chronic TBI pathology is not yet fully understood. Previous studies showed that the terminal component of the complement system (membrane attack complex [MAC]) is the major mediator of damage [39]. Here, we found that multiple members of this system are acutely and chronically dysregulated across multiple post-injury intervals in a coordinated manner (Fig 7A and Fig 7B), which is evidence for a causal role for this system in chronic neurodegeneration.

**Fig 8:**
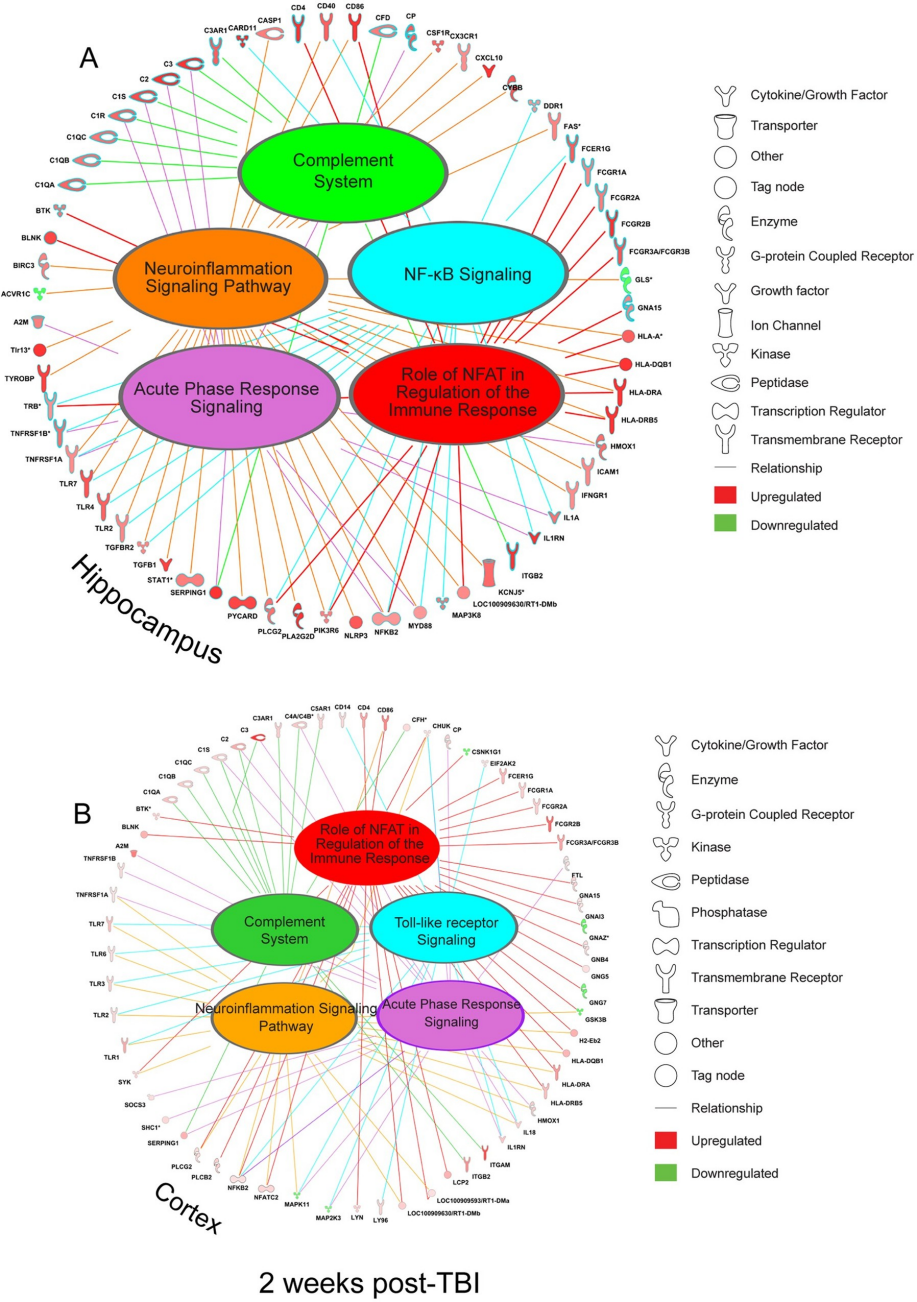
Ingenuity pathway analysis of gene expression at 2 weeks post-TBI. At 2 weeks post-injury, there is a sustained and persistent upregulation of genes in the most of the same immunoregulatorypathways identified at 24 hr post-injury in the hippocampus (A) and in the cortex (B). Immune related gene expression in cortex is increased vs 24 hr post-injury interval. Many of the differentially expressed genes are key hub genes in essential cell signaling pathways.

At 2 weeks post-TBI, in both the hippocampus and cortex, IPA analyses showed a clustering of dysregulated genes in many of the same immune response pathways found altered at 24 hr (Fig 8A and Fig 8B). Notably, genes in the complement system remain strongly activated. What is striking, however, is the increase in cortical expression of many immunoregulatory genes that were not significantly expressed at 24 hr in the cortex. Given that immune response has both beneficial and deleterious components, we speculate that some of these delayed gene alterations might be part of a coordinated protective response. For instance, immune mediators such as NF-κB as well as other major transcription factors regulate both pathogenic and prosurvival signaling after TBI [40, 41]. We recently showed that pro-survival genes are downregulated in dying, degenerating hippocampal neurons, including the endogenous neurotrophic molecule *Bdnf* [18]. The NF-κB complex is an upstream regulator which influences the expression of *Bdnf* [42].

Among other immune related processes is leukocyte extravasation [43]. It has been previously reported that blood brain barrier damage and leukocyte infiltration into TBI affected brain parenchyma occur concomitantly up to 72 hr after FPI [44]. While the inflammatory response after TBI often includes the infiltration of peripheral leukocytes in coordination with BBB permeability [45], it has been unclear the extent to which BBB permeability persists and the role leukocyte populations play in continuing neurodegeneration after injury. The persistence of leukocyte extravasation 2 weeks after injury has implications for the post-injury recovery process. Upregulation of leukocyte related genes such as *Cd44*, *Itgb2*, *Ncf1*, and *Rap1b* 2 weeks post-injury indicates a persistent activation of immune cell populations after injury. One of these genes which codes for the hyaluran receptor protein CD44, a cell-surface glycoprotein involved in immune cell adhesion and migration, has been previously shown to be upregulated up to three months after traumatic injury [46, 47]. *In silico* analysis indicated that the multiple immune-related genes upregulated in the hippocampus and cortex at 2 weeks are all implicated in critical cell functions. For instance, *Rap1b* has essential roles in immune function [48] and in angiogenesis and endothelial permeability [49]. This hints at a regenerative response that is reflected in the long-term chronic data.

Custom PCR array analysis at one and two months post-TBI was helpful in filling in gene expression trends observed at 2 weeks post-injury (Table 1 lists significantly expressed genes in hippocampus and cortex at one month post-injury). At one month post-TBI, in both the hippocampus and cortex, several genes that are involved in inflammation and immune resonse were still significantly upregulated. However by 2 months post-TBI we find that these same genes are upregulated but no longer significantly different between TBI and sham controls. The complete list of PCR array genes with their annotations are provided in Supporting Information S1 Table and complete PCR array results are provided in S2 Table.

**Table 1:** Custom PCR array analysis of significant gene expression changes in hippocampus and cortex one month post-injury.

Symbol	Gene Name	Fold Change (TBI relative to Sham)	Tukey's (p-value <0.05) (TBI vs. Sham)	Fold Change (TBI relative to Sham)	Tukey's (p-value <0.05) (TBI vs. Sham)
		Hippocampus		Cortex	
A2M	alpha-2-macroglobulin	2.739604343	0.000567200	4.182845172	0.000354900
BCL3	B-cell CLL/lymphoma 3	6.482178181	0.006074100		
BCL2A1	BCL2 related protein A1	2.440369625	0.011243700	1.454297076	0.000265900
BDNF	brain-derived neurotrophic factor			0.629889605	0.032091000
CASP4	caspase 4	2.581907185	0.009087000	1.004270534	0.000698500
CD44	CD44 molecule (Indian blood group)	2.362791778	0.001476900	1.710251855	0.007365700
CD86	CD86 molecule	3.278563714	0.007453900	2.332680942	0.000910400
CP	Ceruloplasmin	3.724613546	0.024728700	1.367472055	0.004890400
CCL2	chemokine (C-C motif) ligand 2	4.388632897	0.022398100		
CCL4	chemokine (C-C motif) ligand 4	8.927629673	0.002840700	1.346820049	0.005942400
CXCL10	chemokine (C-X-C motif) ligand 10	8.562155713	0.006765800	3.655353772	0.008301600
C2	complement component 2	4.825954831	0.000012100	1.907245523	0.000002500
C3	complement component 3	11.895379933	0.000780600	2.877940365	0.000440800
Clec4a2	C-type lectin domain family 4, member A2	2.242857802	0.006765400	2.834788106	0.002065900
FCGR2B	Fc fragment of IgG receptor IIb	4.342347521	0.001543100	1.649755706	0.000211600
LGALS3	galectin 3	7.666670000	0.009318700	11.345113604	0.020632200
Hmox1	heme oxygenase 1			1.925523325	0.043166200
ITGB2	integrin subunit beta 2			3.847594168	0.017574000
IRF8	interferon regulatory factor 8	2.895076949	0.006587400	2.927772338	0.002035000
NCF1	neutrophil cytosolic factor 1	4.278083111	0.000991600	2.329630296	0.000608100
PLD4	phospholipase D family member 4	2.532655728	0.007464100	1.768078558	0.001902900
PARP9	poly(ADP-ribose) polymerase family member 9			0.841178319	0.121326000
RAC2	ras-related C3 botulinum toxin substrate 2 (rho family, small GTP binding protein Rac2)	2.428224016	0.015031300	2.396043983	0.004905400
ARHGDIB	Rho GDP dissociation inhibitor (GDI) beta			0.850419421	0.001774400
S100a6	S100 calcium binding protien A6	2.760251556	0.015059200		
SPP1	secreted phosphoprotien 1	9.084560674	0.000718100	9.791226242	0.003217900
TLR4	toll-like receptor 4			1.338358759	0.003078200
TGFB1	transforming growth factor beta 1	2.552758483	0.006375400	1.150126262	0.033726000
TUBB6	tubulin beta 6 class V			1.712198571	0.028528600

Genes were selected following Ingenuity Pathway Analysis of acute and chronic hippocampal and cortex microarray data. Curated information on all custom PCR array genes including significant expression at post-injury intervals, GeneCard links and relevant PubMed links shown in Table 1 and complete custom array results for hippocampus and cortex shown in S1 Table and S2 Table.

At chronic post-injury intervals, we found fewer canonical pathways are significantly activated or inhibited. However, we found that dysregulated genes in the complement pathways, C2 and C3 specifically, are consistently upregulated across multiple time points as well as the “Acute Phase Response” and “Leukocyte Extravasation” pathways which remain activated. Again as in earlier time points, several pro-survival genes are upregulated at chronic post-injury intervals. For instance, increased levels of *Bcl-3*, a prosurvival gene [50], is only detected 3 months post-injury as is *S100A6* [51]. A previous study showed that a decrease in *S100A6* expression in the rat hippocampus is associated with TBI-induced cognitive deficits [52]; therefore, the increased expression at 3 month suggests a regenerative response. SPP1, aka osteopontin, a cytokine with neuroprotective [53] and regenerative effects [54] is also upregulated at 3 months.

At 6 months post-injury, genes in “Complement System”; “Acute Phase Response Signaling”; and, “IL-6 Signaling” were still dysregulated. Core components of the complement cascade, i.e. C2 and C3, continue to be upregulated in the hippocampus. Genes involved in cytokine signaling, *Il-1β* and *Il-6*, also remain elevated at this time point.

At one year post-injury, there were no significantly altered pathways which were overtly related to immune-related function. However, we identified dysregulated genes that are involved in both immune cell signaling, such as the interleukin family of genes and neurotransmitter related pathways (“cAMP-Mediated Signaling”; “Axonal Guidance Signaling”; and “Semaphorin Signaling in Neurons”) such as the dopamine receptors (DR), *Drd1* and *Drd2*. Importantly, at one year post-injury, many of the genes in TBI-altered pathways (S1–S4 Files) are known to be involved in neuronal homeostasis and essential brain functions. At the one year post injury time point the most important finding is the differential expression of neurotransmitter related pathways and axonal guidance signaling. This points directly to a regenererative/restorative process that is taking place in the brain long term after TBI.

### Regulator effects analysis in IPA suggests TBI-induced dysregulation of genes involved in cell death and survival

**Fig 9:**
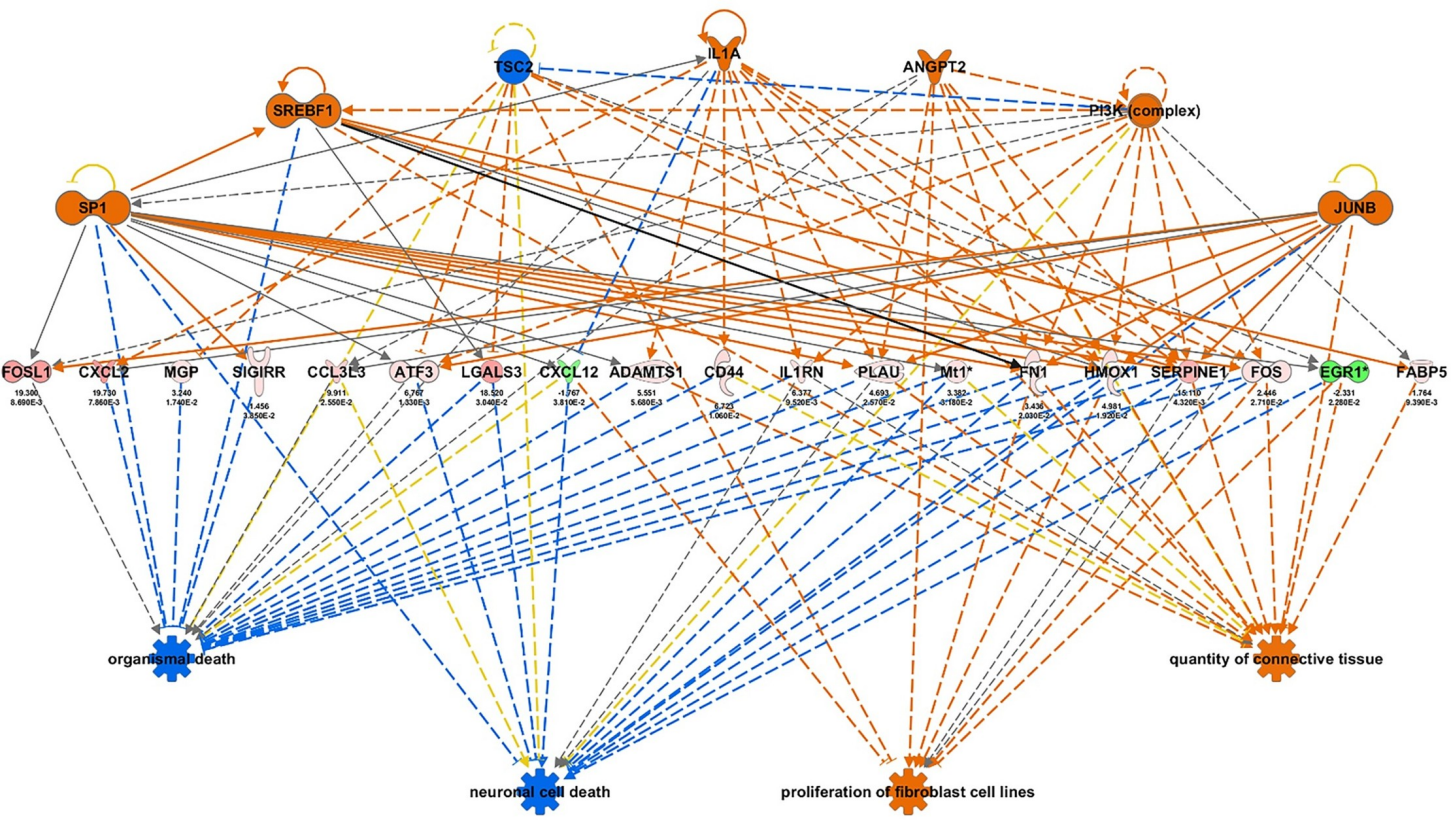
Regulator effects hippocampal network in ingenuity pathway analysis (IPA). Example of integrated results from Upstream regulator and Downstream effects analyses in IPA indicating potential phenotypes or biological impact of TBI-dysregulated genes at 24 hr after TBI in hippocampus. Upstream genes are key hub genes and transcriptional regulators in essential cell signaling networks.

**Fig 10:**
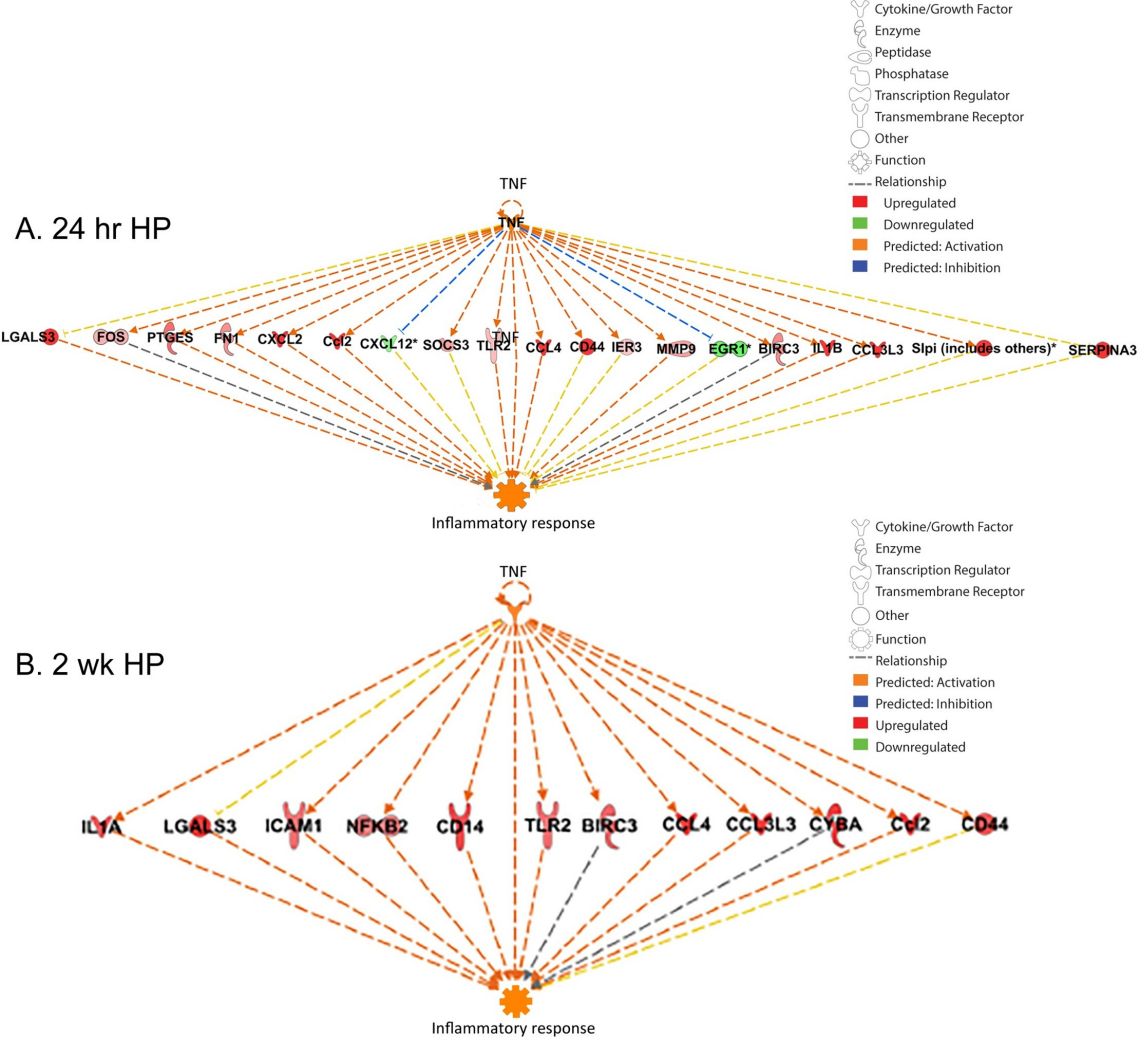
Upstream regulator analysis in ipa identifying tumor necrosis alpha (TNF-α) as a major regulator of TBI-Dysregulated genes in the hippocampus. Upstream regulator analysis of hippocampal gene expression at 24 hr post-TBI (A). Upstream regulator analysis shows persistent dysregulation of genes predicted to be regulated by TNF-α 2 weeks post-injury in hippocampus (B).

IPA’s Regulator Effects analysis tool allows interactive modeling of upstream and downstream relationships in our dataset. By integrating results from Upstream regulator and Downstream effects tools, we can create hypotheses that explain what is going on upstream to the experimentally measured gene changes that are linked to a phenotype or other functional outcomes. For instance, regulator effects analysis (Fig 9) shows all predicted upstream genes for hippocampal TBI-induced gene changes at 24 hr are key hub genes that have essential roles in cell function and disease [55–57]. Moreover, many of the genes we find significantly altered by TBI, such as *Atf3* [58], are also hub genes and/or transcriptional regulators of essential cell functions. Importantly, the downstream effects tool which would predict the biological impacts of upstream molecules based solely on the activity of the TBI-induced gene sets, shows the functional roles of these genes in neuronal cell death, organismal death and notably, cell proliferation and survival, hinting at an early regenerative response. This regenerative response is reflected in the expression of axon guidance genes at one year after injury. We also observed that with longer post-injury intervals, there were increased expression of other known protective genes (i.e. *Creb5*, *NgfR* and *Stat3*), which merit closer examination in future studies. Upstream regulator analysis also allows us to identify and explore the interactions of individual hub genes that have biological signicance in TBI. For instance, at both 24 hr and 2 week post-TBI in the hippocampus, we identified TNF as a key regulator of multiple differentially expressed immune-related genes that are implicated in the inflammatory response (Fig 10).

## Discussion

The key findings of this study are 1) evidence of long-lasting dysregulation of homeostatic gene expression, particularly immunoregulatory gene expression; 2) many dysregulated genes are key hub genes involved in essential brain cell functions (i.e. metabolism, cell morphology, synaptic function, proteostasis); and 3) the chronic activation of both deleterious and beneficial genes up to a year post-injury suggests the existence of a potentially wider therapeutic window than previously indicated.

The lack of therapeutic interventions is a longstanding frustration for those who treat TBI. In this regard, a compelling reason for our focus on persistent inflammatory dysfunction is that simply documenting chronic neuropathological changes after TBI is not enough; we need to identify druggable molecular targets in chronic TBI so that we can explore therapeutic options that can be immediately implemented in the existing population of TBI survivors. The persistent, long-term dysregulation of immunoregulatory genes in TBI rats is suggestive of a much wider therapeutic window than previously thought and implies that treatment of chronic TBI survivors with immunomodulatory drugs may be beneficial months to years after their injuries.

Neuroinflammation has been extensively documented in animal models of TBI, but with a few minor exceptions, most studies only examined inflammatory effects days to a month only after injury [59]. Our findings are consistent with considerable existing evidence showing that uncontrolled and persistent inflammation is a driver of many progressive neurodegenerative diseases [60, 61]. One clue to the causal link between chronic inflammation and neurodegeneration is that the immune mediators found dysregulated in our study have been shown to regulate or influence key genes essential for protein homeostasis (proteostasis), genes that are also found dysregulated in neurodegenerative diseases [19, 62]. These findings are also concordant with the significant finding of our recent study of microRNA regulation in rat TBI [18], that TBI-induced miRNAs suppress hundreds of target genes essential for proteostasis. Moreover, analysis of the OMIM (Online Mendelian Inheritance in Man) database shows that the differentially expressed genes identified in this study are involved in many known human diseases.

One aspect of brain injury that has troublesome implications for the global burden of disease and is insufficiently understood is that TBI itself is a well documented risk factor for age-related neurodegenerative disorders such as AD and PD and psychiatric disorders such as depression [6, 8]. The role that chronic inflammation plays in the pathogenesis of aging and aging-related neurodegenerative diseases such as AD is well established [63, 64]. Thus, the chronic dysregulation of immune related genes in our study provides a mechanistic rationale for how TBI-induced inflammation increases the risk of age-related brain diseases after TBI [65]. Likewise, since chronic inflammation is also causally linked to depression [66], the sustained and persistent inflammatory response in TBI rats is another piece of evidence linking TBI with increased risk of depression. Brain injury induced inflammation is also thought to contribute to the development of neurologic disorders such as epilepsy [67]. This may relate to the activation of NFκB which has been shown to affect synaptic channel function and activity [68]. Moreover, a common upstream regulator found upregulated at 24 hr and 2week, TNF-α, has been shown through computational modeling to modulate synaptic scaling, thus contributing to epileptogenesis [69]. Together, our study suggests that abnormal expression of immune mediators and brain injury-induced factors may be involved in the etiology of various neurologic and neurodegenerative comorbidities of TBI.

It has long been thought that failure to regenerate is a major cause of neurodegeneration [70] and we have shown that TBI induces multiple genes and pathways involved in regeneration and survival as well as neurodegeneration [17]. Since cytokines have both pro and anti-inflammatory effects which would impact regenerative processes [71], the expression of regenerative immunoregulatory genes at chronic postinjury intervals (i.e. *Ngfr*, *Stat3*, *Il-18*, *HspB1*) may provide additional targets for therapeutic modulation. Increased levels of dopamine receptors correlate with brain plasticity [72]. Thus, the sustained expression of these neuroplasticity genes in the hippocampus is another indication of a long-term regenerative response. There is an important therapeutic implication here; evidence of a protective, regenerative response at all post-injury intervals indicates that therapies augmenting these responses may be beneficial regardless of how many months or years have passed since the initial TBI. For instance, since steroid-induced decreases in *S100b* levels have been shown to reduce inflammation in the hippocampus in a rat model of epilepsy, the significant decrease in expression of *S100b* at one year post-TBI would protect against inflammation [73]. The increased expression of *Atf3* also appears to be a protective response (see Fig 6); a recent study found that neuroinflammation was enhanced in *Atf3* knockout mice after TBI [74]. Our study reinforces the idea that some components of the inflammatory response are beneficial and indeed, essential for recovery [60]. We previously observed that a strong regenerative response involving developmental and neurotransmitter genes is activated in surviving brain cells after TBI [17]. Overall, our long-term gene expression data is consistent with recent reports that describe therapeutic strategies to enhance endogenous repair responses [75]. Inhibition of MAC formation in mice reduced axonal loss as well as mitigated the accumulation of microglia and apoptotic signaling [76]. Given evidence that complement proteins are involved in synaptic degeneration [77] and inhibition of complement increases expression of genes involved in neuroplasticity [78], our finding that core components of the complement cascade are upregulated in hippocampal tissue as long as 6 months after injury provides a mechanistic rationale linking disruption of neuroplasticity with neurodegeneration. On the other hand, the increase in genes involved in neurotransmission and axon guidance at one year suggests that homeostatic regenerative responses have stabilized the injured brain by this time.

Although wholly speculative, there are translational implications of this study; for instance FDA-approved anti-inflammatory drugs [79] such as edavarone, have demonstrated neuroprotective potential [80]. The anti-diabetic drug, metformin, also has neuroprotective effects via activation of antioxidant and anti-inflammatory pathways [81]. Since antidepressants share common molecular mechanisms with anti-inflammatory drugs [82], these common mechanisms appear to be the basis for the ability of antidepressants to protect against neurodegeneration [83]. Given that TBI is a risk factor for depression [84] and TBI patients with depression are treated with antidepressant drugs, we speculate that the recovery of the TBI patients treated with antidepressants may be attributed, in part, to the anti-inflammatory effects of these drugs [85].

One of the most studied genes in the human genome, TNF-α [86], is a key upstream regulator of many immunoregulatory genes in our study. There are several approved drugs targeting TNF-α; for instance, some inhibitors of TNF-α such as the thalidomide analogue 3,6’-dithiothalidomide (DT) have been shown to reverse hippocampal-dependent cognitive deficits- by restoring neuronal function and reducing the effects of inflammation- induced by chronic inflammation in rats [87]. The neuroprotective properties of omega-3 fatty acids (FA) in fish oil are thought to be mediated by the ameliorating effects of FA on TLR-induced inflammation [88]. Omega-3 FA and aspirin have also been shown to modulate microglial mediated inflammation and may provide protection against brain diseases with inflammatory etiology [89]. Interestingly, given that exercise pre-conditioning reduced brain inflammation and protected against TBI in rats [90], it is notable that exercise is known to improve cognitive performance in people with mild or moderate TBI [91]. The existence of chronic therapeutic windows is also supported by a mouse study showing that late exercise (5 weeks after TBI) but not early (1 week after TBI) reduced inflammation and cognitive dysfunction after TBI [92].

The limitations of this study were, in part, due to constraints imposed by the larger, comprehensive analysis of chronic TBI. Only a fraction, 4 of 12 rats per experimental group, were available to be processed for microarray analysis. The remaining rats were processed for parallel proteomic and histopathological analyses. Eventually, the genomic data will be compared across all time points with the proteomic and histological data and this comprehensive analysis is expected to shed significant light on the chronic disruption of brain functions after TBI. However, for this study, our confidence in the biological relevance of the chronic data is largely supported by the results of our recent genomic studies as well as extensive *in silico* validation of the differentially expressed genes.

## Supporting information

S1 Fig Principal component analysis (PCA) of hippocampal gene expression in naïve, sham control and traumatic brain injured (TBI) rats at 24 hour, 2 weeks, 3 and 6 months post-injury. (TIF)

S2 Fig Principal component analysis (PCA) of cortex gene expression in niave, sham control and traumatic brain injured (TBI) rats at 24 hour, 2 weeks, 3 and 6 months post-injury. (TIF)

S3 Fig Quantitative PCR validation of hippocampal gene expression microarray data from five acute and chronic post-injury intervals (24 hour, 2 weeks, 3, 6 and 12 months). (TIF)

S4 Fig Quantitative PCR validation of cortex gene expression microarray data from five acute and chronic post-injury intervals (24 hour, 2 weeks, 3, 6 and 12 months). (TIF)

S1 File TBI-associated genes (absolute fold-change >1.4, p < 0.05, TBI vs sham control) in all significant canonical pathways in hippocampus. All time points are shown in separate tabs.
(XLSX)

S2 File TBI-associated genes (absolute fold-change >1.4, p< 0.05, TBI vs sham control) in all significant canonical pathways in cortex. All time points are shown in separate tabs.
(XLSX)

S3 File TBI-associated genes in all significant diseases and functions pathways in hippocampus. Genes for each post-injury interval listed in separate tabs.
(XLSX)

S4 File TBI-associated genes in all significant diseases and functions pathways in cortex. Genes for each post-injury interval listed in separate tabs.
(XLSX)

S5 File Gene Ontology (GO) categories for post-TBI hippocampal gene expression. GO categories for 24hr, 2 week, 3, 6, 12 month post-TBI listed in separate tabs.
(XLSX)

S6 File Gene Ontology (GO) categories for post-TBI cortex gene expression. GO categories for 24hr, 2 week, 3, 6, 12 month post-TBI listed in separate tabs.
(XLSX)

S1 Table List of 84 genes selected for a custom PCR array. (XLSX)

S2 Table Complete custom PCR array results for hippocampus and cortex at one and two months post-injury (separate tabs for each brain region and post-injury interval). (XLSX)
