# Supplementary figures and images for: Traumatic brain injury induces long-lasting changes in immune and regenerative signaling
Source: PLoS One. 2019 Apr 3;14(4):e0214741. doi: 10.1371/journal.pone.0214741 (PMC6447179; doi:10.1371/journal.pone.0214741)

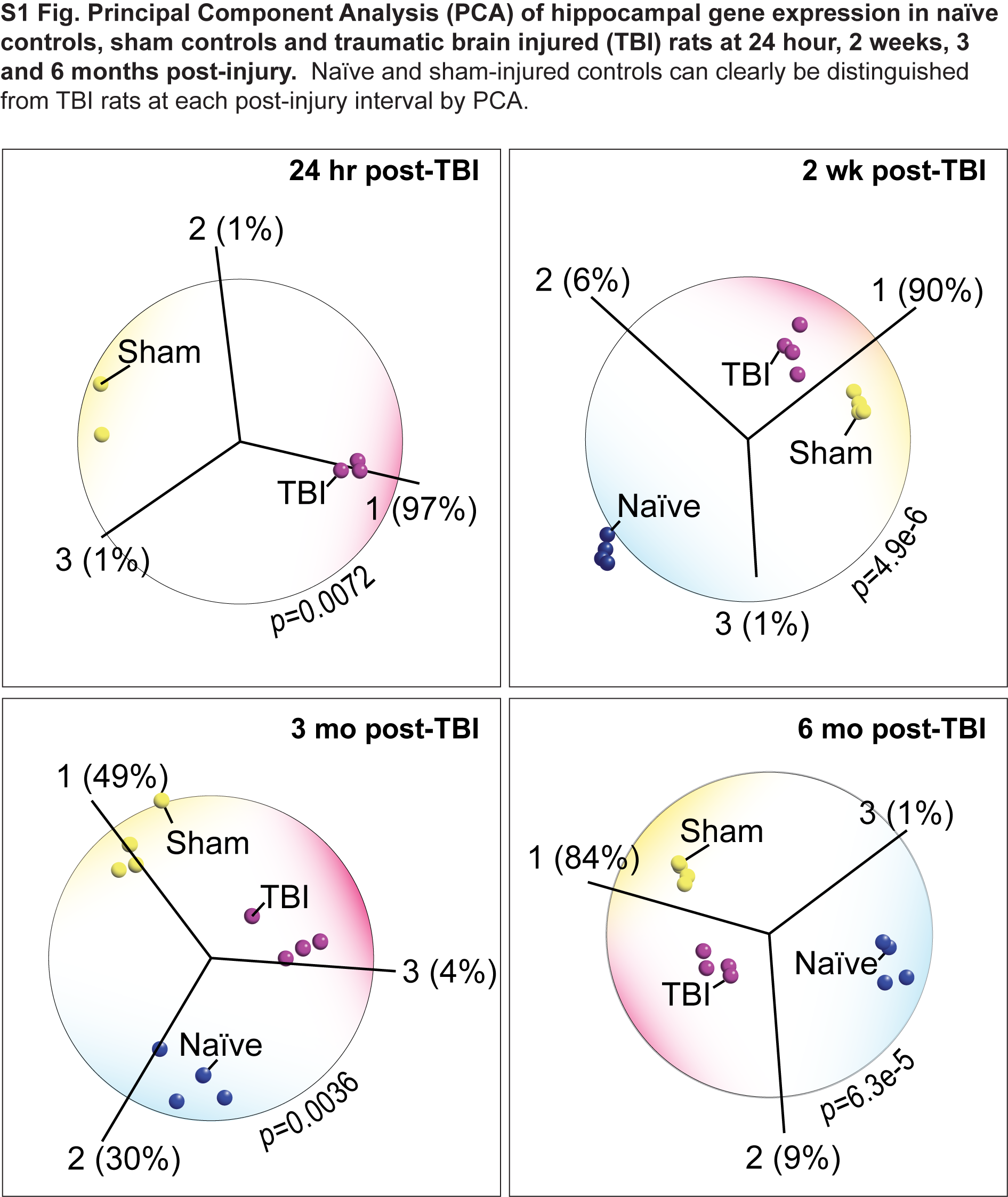

Supplement: S1 Fig — (TIF) [file pone.0214741.s001.tif]

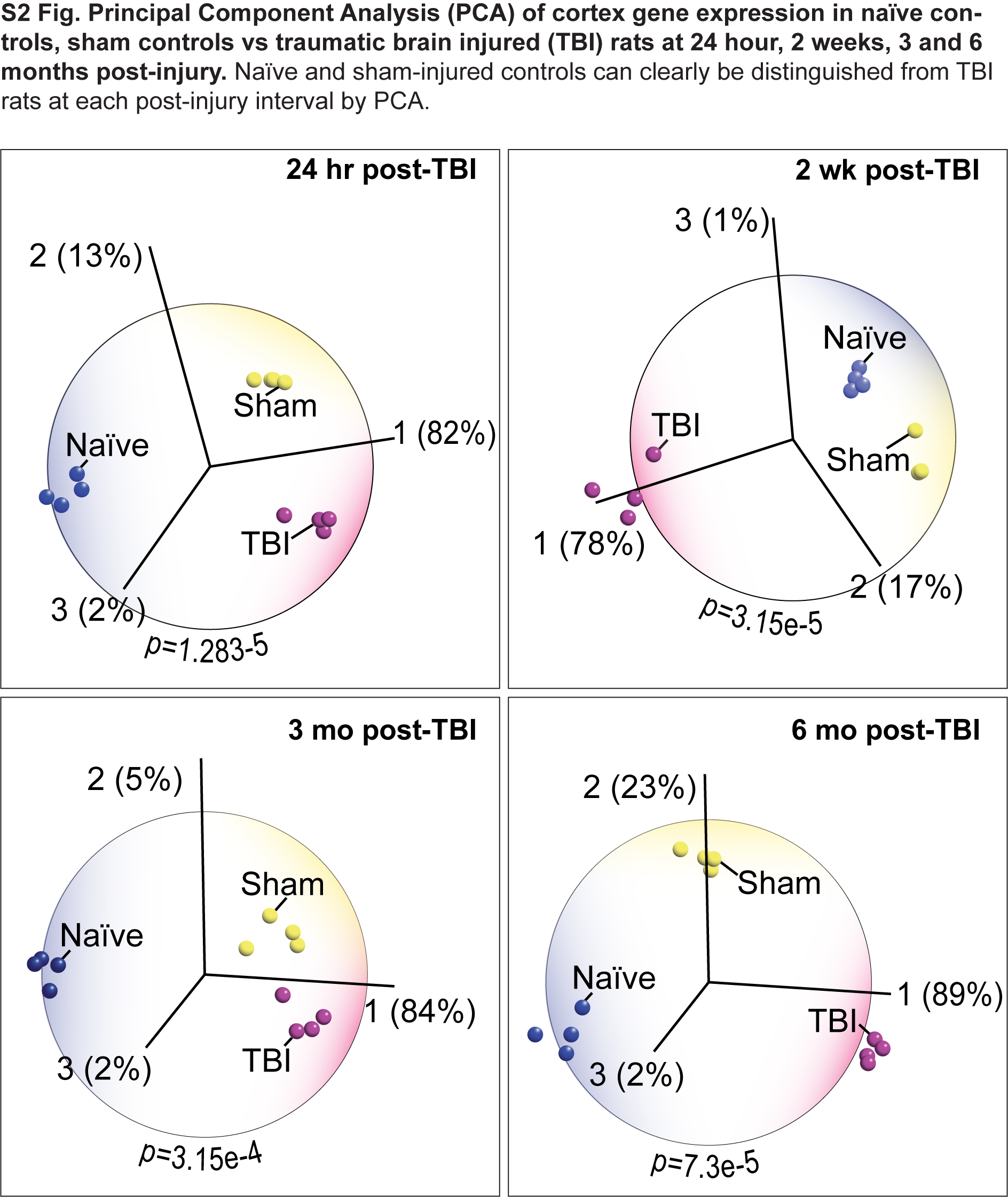

Supplement: S2 Fig — (TIF) [file pone.0214741.s002.tif]

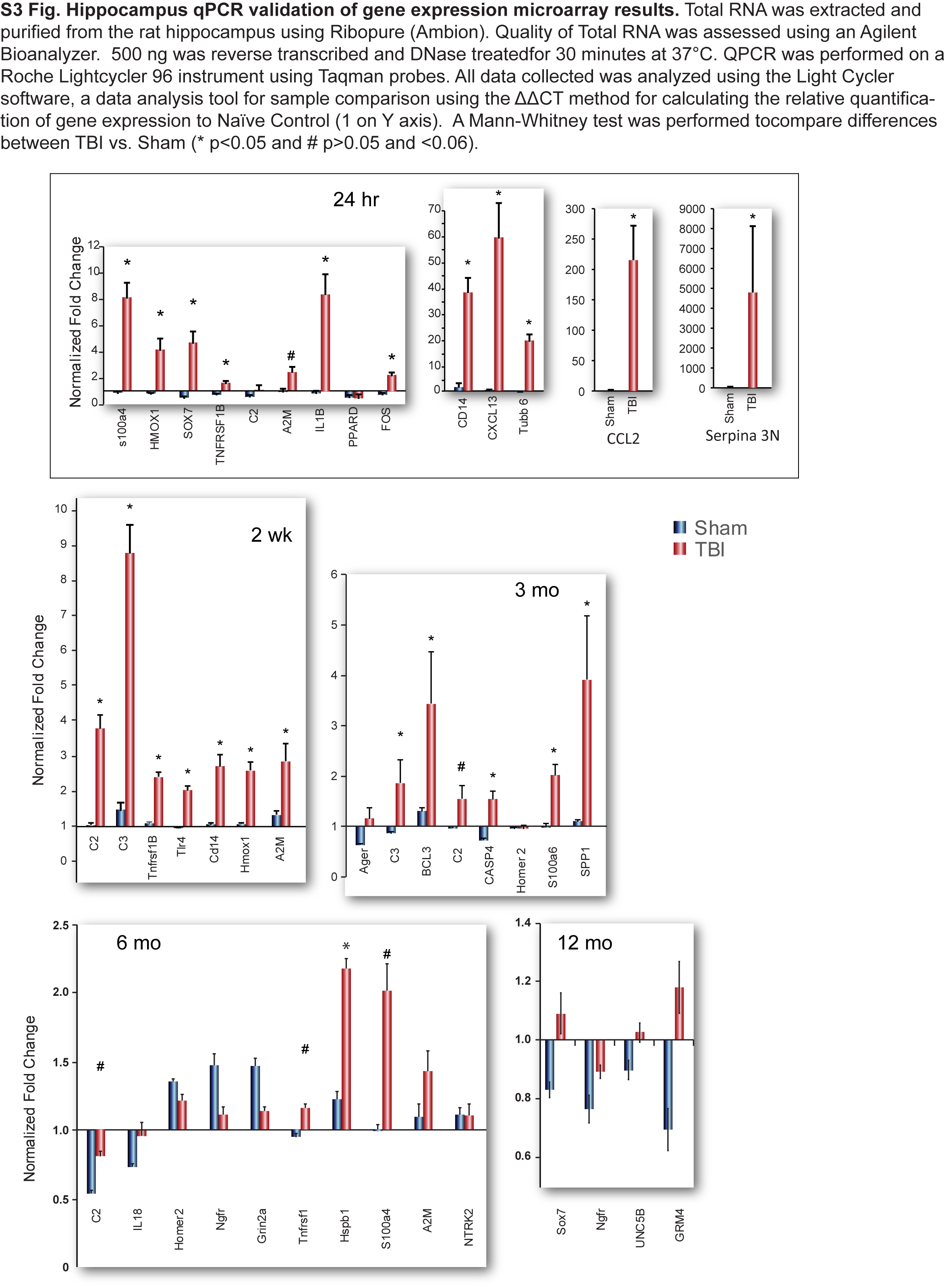

Supplement: S3 Fig — (TIF) [file pone.0214741.s003.tif]

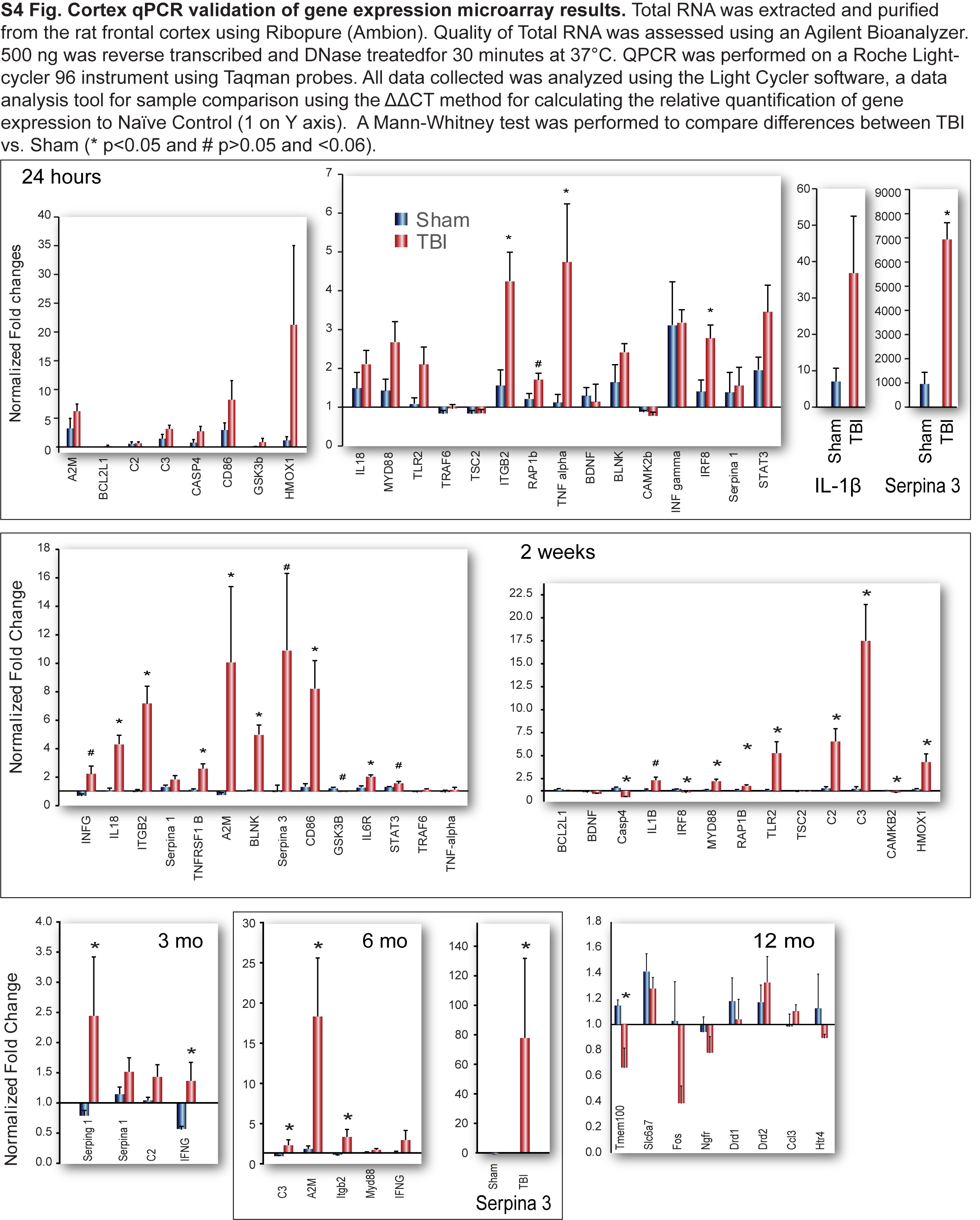

Supplement: S4 Fig — (TIF) [file pone.0214741.s004.tif]
